# Comparison of the Efficacy of D2 Gastrectomy Plus Liver Radiofrequency Combined With Chemotherapy Versus Chemotherapy Alone in the Treatment of Advanced Gastric Cancer With Unresectable Synchronous Liver Metastases: A Multicenter Randomized Controlled Trial Protocol

**DOI:** 10.3389/fonc.2022.802683

**Published:** 2022-02-28

**Authors:** Weidong Wang, Ruiqi Gao, Pengfei Yu, Zhenchang Mo, Danhong Dong, Xisheng Yang, Xiaohua Li, Gang Ji

**Affiliations:** Department of Digestive Surgery, Xijing Hospital, Air Force Military Medical University, Xi’an, China

**Keywords:** gastric cancer, synchronous liver metastasis, chemotherapy, radiofrequency, protocol

## Abstract

**Background:**

Whether patients with advanced gastric cancer with unresectable synchronous liver metastases require surgical treatment remains a controversial topic among surgeons. Recently, an open-label multicenter, international RCT study show that compared with chemotherapy alone, gastric resection combined with chemotherapy had no survival advantage for advanced gastric cancer with unresectable synchronous liver metastases. A limitation of this study was that gastrectomy for gastric cancers was restricted to D1 lymphadenectomy and no metastatic lesions were removed. Whether D2 gastrectomy plus liver radiofrequency plus postoperative chemotherapy could provide benefits to these patients is worthy of further confirmation by high-level evidence-based medicine.

**Methods/Design:**

This study will investigate the efficacy of D2 gastrectomy plus liver radiofrequency plus postoperative chemotherapy compared to chemotherapy alone in a prospective, multicenter, randomized controlled trial that will enroll 200 patients who have advanced gastric cancer with unresectable synchronous liver metastases. The patients will be randomly divided into two groups: the test group (D2 gastrectomy plus liver radiofrequency plus postoperative chemotherapy, n=100) and the control group (chemotherapy alone, n=100). The patients’ general information, past medical history, laboratory tests, imaging results, surgery details, and chemotherapy details will be recorded and analysed. The overall survival (OS) will be recorded as primary endpoints. Progression-free survival (PFS) and the total incidence of complications will be recorded as secondary endpoints.

**Discussion:**

This study is to establish a multicentre randomized controlled trial to compare the efficacy of D2 gastrectomy plus liver radiofrequency combined with postoperative chemotherapy versus chemotherapy alone.

**Trial Registration:**

Chinese Clinical Trial Registry, Approved No. of ethics committee:ChiECRCT20200331. Registered on 15 November 2020. Registration number:ChiCTR2000039964. The study has received full ethical and institutional approval.

**Advantages and Limitations of this Study:**

This is the first clinical trial that will provide evidence on the efficacy of D2 gastrectomy plus liver radiofrequency combined with chemotherapy versus chemotherapy alone for the treatment of advanced gastric cancer with unresectable synchronous liver metastases. A prospective RCT with 200 patients who have advanced gastric cancer with unresectable synchronous liver metastases.

**Clinical Trial Registration:**

[https://www.chictr.org.cn/], identifier ChiCTR2000039964.

## Background

Gastric cancer (GC) ranks fifth in incidence and third in mortality among all cancers worldwide each year (1). Synchronous liver metastasis occurs in 3%-14% of GC patients and has a very poor prognosis (2, 3). Currently, chemotherapy is recommended as the standard treatment for advanced gastric cancer with unresectable synchronous liver metastasis (GCLM) by Japanese, American, and international guidelines (4, 5). Although the therapeutic effect has been improved to a certain extent due to the continuous improvement in chemotherapy in the past decade, patients with GCLM have a poor prognosis under this treatment (6, 7). Therefore, there is an urgent need for a new therapeutic strategy to improve survival and prognosis in patients with GCLM.

Previous studies (8–10) showed that palliative gastrectomy combined with chemotherapy could improve the survival of patients with GCLM. However, a recent multicenter randomized controlled study (11) published in Lancet Oncology reported that gastrectomy plus chemotherapy did not provide a survival advantage in the treatment of GCLM compared with chemotherapy alone. However, the method of GC surgery in this study was D1 lymphadenectomy in gastrectomy for primary gastric tumors, which was performed in patients who received chemotherapy after gastrectomy. The surgical methods used in our study will be D2 lymphadenectomy in gastrectomy for primary gastric tumors, a more thorough dissection for the possible occurrence of lymph node metastasis in patients with GC.

Most of the current clinical studies are retrospective cohort studies (12, 13), so the current evidence is not sufficient to support a treatment strategy of D2 gastrectomy plus liver radiofrequency plus postoperative chemotherapy for standardized treatment of patients with GCLM. At the same time, there is also a lack of prospective studies on the efficacy of D2 gastrectomy plus liver radiofrequency plus postoperative chemotherapy versus chemotherapy alone for treatment in China. If the results obtained in this study are in line with our expectations, this study will supplement the existing deficiency of treatment regimens for GCLM, which is of great significance for improving the prognosis of patients with GCLM in clinical practice, and even for the development of a new, more feasible and effective standardized treatment strategy for patients with GCLM.

## Methods/Design

**Figure 1:**
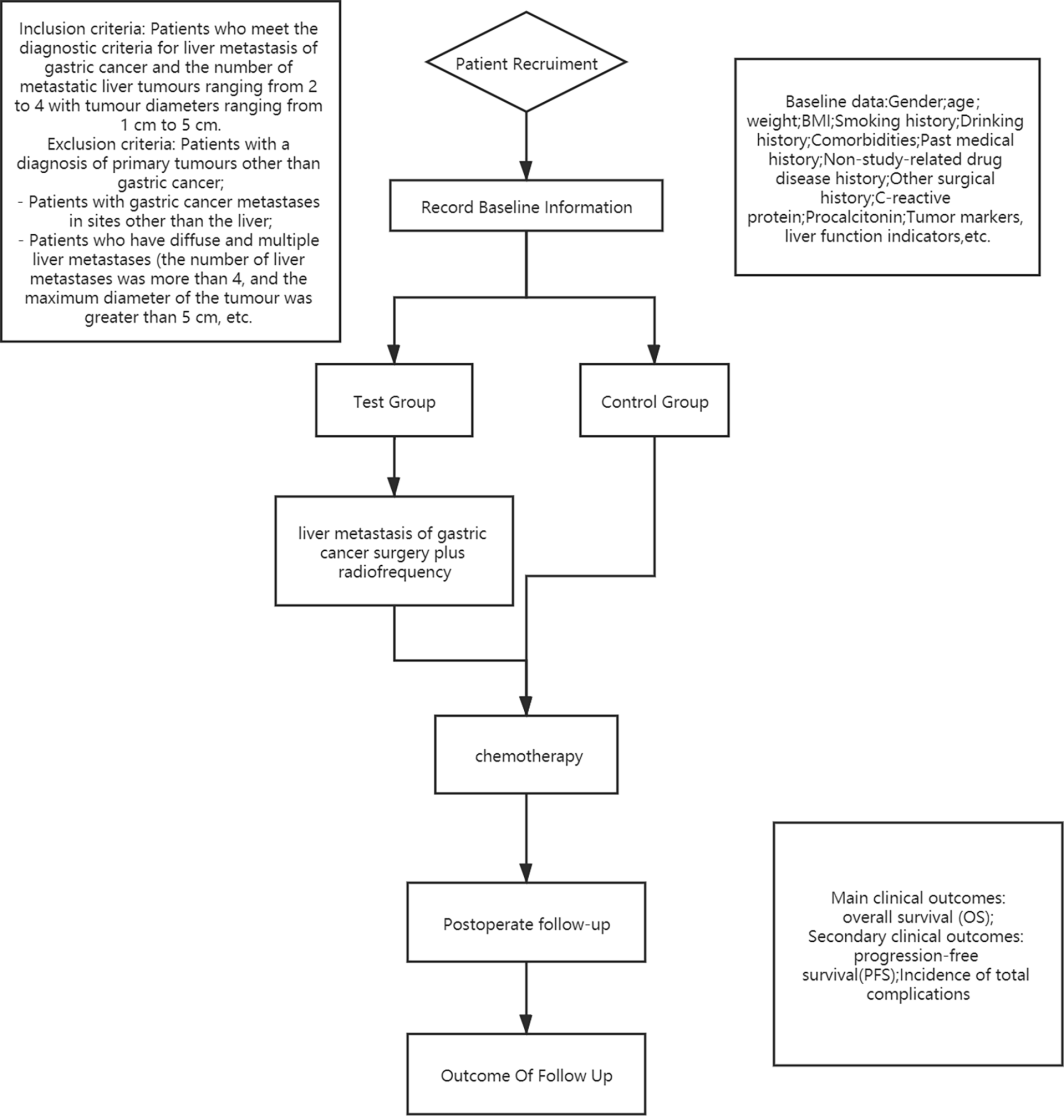
This is the whole flow diagram of the test.

This study will be a multicenter RCT in which 200 patients will be enrolled from November 2020 to November 2022. They will be randomly designated to the test group or the control group in a 1:1 distribution ratio. The test flow chart is shown in **Figure 1**.

### Main Objective

According to the process shown in **Figure 1**, 200 patients with GCLM will be grouped to explore a comparison of the OS of D2 gastrectomy plus liver radiofrequency plus postoperative chemotherapy versus chemotherapy alone.

### Secondary Objectives

- To compare the PFS of patients in the treatment of D2 gastrectomy plus liver radiofrequency plus postoperative chemotherapy to those treated with chemotherapy alone

- To explore a comparison of the total incidence of complications of D2 gastrectomy plus liver radiofrequency plus postoperative chemotherapy to those of chemotherapy alone

### Patient Recruitment and Characteristics

The recruitment method will be to collect patients during routine procedures who were screened by investigators against the exclusion criteria. The researchers will have them sign the informed consent form. The above procedures conform to the provisions of the Measures for Ethical Review of Biomedical Research Involving Human Beings (Trial), the Declaration of Helsinki v.08 and the International Ethical Guidelines for Biomedical Research Involving Human Beings.

### Inclusion Criteria

Patients will be included when they meet all of the following conditions:

- Patients who are male or female, aged 20 to 75 years;
- Patients who were diagnosed histologically with primary gastric adenocarcinoma;
- Patients diagnosed with clinical T1–3 disease by laparotomy or laparoscopy (for T1-T3 patients with the node positive, in order to eliminate the inter group differences, they were randomly divided into control group or experimental group according to the clinical nodal status (further subgroup: N0–1 vs N2–3). For patients with para-aortic lymph node metastasis above the coeliac axis or below the inferior mesenteric artery (lymph node 16a1/b2 of maximum diameter ≥1 cm), or both, or T4 patients with the node positive, according to the NCCN diagnosis and treatment guidelines for gastric cancer and the recommendations of our multidisciplinary diagnosis and treatment team, neoadjuvant therapy was first chosen) (11);
- Patients who will undergo diagnostic laparoscopic exploration combined with peritoneal lavage cytology to exclude peritoneal disseminated implant metastasis that is not visible to the naked eye (14);
- Patients who will be examined for the molecular profile of the tumors (HER2, PDL1, MSI and MMR expression) [patients with HER2-positive advanced gastric cancer were excluded since trastuzumab in combination with chemotherapy has become the standard treatment for these patients (15)];
- Patients with distant metastases that were limited to synchronous liver metastases confirmed by both laparotomy and CT scan;
- Patients with metastatic liver tumor ranging from 2 to 4 with tumor diameters ranging from 1 cm to 5 cm (11) (In order to avoid the omission of potential liver metastases, we used contrast-enhanced liver ultrasound as one of the means of further screening. Considered that unless data are now available from ongoing trials, ablation for resectable liver metastasis lesions should not be used in radiofrequency of liver resection. We recommend all patients to multidisciplinary tumor boards to determine whether the liver metastasis is actually unresectable);
- Patients whose PS (performance status) was from 0 to 1;
- Patients who have not previously been treated for gastric cancer other than EMR;
- Patients who have not previously received radiation therapy or chemotherapy for any other malignancy;
- Patients who have no contraindications to treatment (surgery, radiofrequency ablation, and chemotherapy);
- Patients and their families voluntarily participated in the study and signed the informed consent form.

### Exclusion Criteria

Patients will be excluded when they meet any of the following conditions:

- Patients with a diagnosis of primary tumors other than gastric cancer;
- Patients who have gastric cancer metastases in sites other than the liver;
- Patients who have coagulation dysfunction that cannot be corrected;
- Patients with viral hepatitis and cirrhosis;
- Patients with diabetes mellitus, uncontrolled or controlled with insulin;
- Patients treated with systemic steroids;
- Patients suffering from psychosis;
- Patients with heart, lung, liver, brain, kidney or other organ failure;
- Patients who have ascites and cachexia preoperatively, and their general conditions are poor;
- Patients who refuse to sign the informed consent to take part in this study;
- Female patients who are during pregnancy or breast-feeding (except for those women who are breast feeding but consenting to surgery and chemotherapy).

### Terminating Study Criteria

The terminating study criteria are as follows:

- Patients are unable to undergo surgery for various reasons after enrollment (reasons need to be recorded);
- The researchers consider that the patients are not suitable to continue the clinical trial (the reason for withdrawal should be recorded);
- Patients with serious complications or unbearable adverse reactions;
- Patients request that the trial be terminated;
- Patients violated the treatment principles (violation of injection and discharge standards, disobedience to the study chemotherapy arrangement, etc.)

We will terminate the study for individual patients once they meet the termination criteria and their data will not be included in the final analysis. Other patients who meet the criteria for admission but do not meet the criteria for termination will continue to participate in our study.

### Participating Entities

This clinical trial is a multicenter study. The institutions are as follows: the First Affiliated Hospital of Air Force Military Medical University, The Second Affiliated Hospital of Air Force Medical University, The First Affiliated Hospital of Xi’an Jiaotong University, Zhongshan Hospital Affiliated to Fudan University and West China Hospital of Sichuan University. The above institutions all have sufficient experience in the diagnosis and treatment of gastrointestinal tumors.

### Randomization Procedure

Eligible participants will be randomly designated to either the test group or the control group in a 1:1 distribution ratio. First, all enrolled patients were tested the molecular profile of the tumors, such as HER2, PDL1, MSI and MMR expression. And after the patient underwent molecular profile testing, patients with HER2-positive advanced gastric cancer were excluded since trastuzumab in combination with chemotherapy has become the standard treatment for these patients. For the rest of the other patients, follow up treatment protocols could be adjusted according to the patients’ molecular profile, such as immunotherapeutic antibody PD-1 or PD-L1, and so on. Accordingly, in the statistical analysis of data, different patients also need to conduct subgroup hierarchical analysis according to the molecular detection results of tumors to reduce data error. Biostatisticians not involved in this study will use SAS software 9.2 (SAS Institute, Cary, NC, USA) to generate random sequences. A research assistant not participating in the recruitment process will seal the random list in sequentially numbered nontransparent envelopes stored in a cabinet that is double-locked. The research assistant will store the randomly assigned envelopes separately. Data collection and data analysis will be blinded, except that the intervention is not blinded to participants and clinicians (16).

### Treatment Protocols

All eligible patients will be randomly divided into a test group or a control group according to a 1:1 distribution ratio. The test group will receive D2 gastrectomy plus liver radiofrequency plus postoperative chemotherapy. In the test group, only open surgery is allowed. The control group will receive chemotherapy alone. The chemotherapy regimens of the two groups are the same (11), S-1 plus cisplatin. S-1 will be given orally 80-120 mg/m² per day for the first 3 weeks of every 5-week cycle. The dose of S-1 will be given according to the patients’ body surface area (less than 1.25 m², 80 mg; 1.25–1.5 m², 100 mg; and greater than 1.5 m², 120 mg). Patients will receive cisplatin 60 mg/m² on Day 8 of every 5-week cycle *via* intravenous infusion. **Table 1** shows the treatments applied in this study. **Table 2** shows the medication and usage in this experiment. Because all enrolled patients are tested for the molecular profile of the tumors, such as HER2, PDL1, MSI and MMR expression, follow up treatment protocols could be adjusted according to the patients’ molecular profile, such as trastuzumab or immunotherapeutic antibody. Accordingly, in the statistical analysis of data, different patients also require conduct subgroup hierarchical analysis according to the molecular detection results of tumors to reduce data error.

**Table 1:** The treatments applied in this study.

	gastrectomy (total gastrectomy, distal or proximal gastrectomy plus D2 lymph node dissection)	liver radiofrequency	chemotherapy (S-1 + cisplatin)
test group	√	√	√
control group	—	—	√

**Table 2:** The medication and usage in this experiment.

Chemotherapy drugs	dose	Timing of the drugs (a total of 35 days/cycle)			
		Day 1-7/cycle	Day 8/cycle	Day 9-21/cycle	Day 22-35/cycle
S-1	80-120 mg/m² (oral)	√	√	√	—
cisplatin	60 mg/m² (intravenous infusion)	—	√	—	—

### Clinical Data

Clinical data from the patients will be obtained by medical staff and recorded on an online electronic platform (http://www.medresman.org.cn) and in the CRF table. The samples will be coded, and the patients’ identity will be known only by the attending physician. The clinical data will include the following: general patient information, past medical history, past surgical history, laboratory examination results, imaging results, surgery details, PFS and OS. The timing and processing of the above recorded contents will be reflected in the CRF table, and the laboratory examinations will mainly assess preoperative and postoperative routine blood, liver function, tumor markers, and inflammatory indicators. The prognosis of GC with liver metastasis (GCLM) was very poor, with a 5-year survival rate of <10% (17). Patients will be assessed at least monthly from baseline for adverse events *via* verbal interview, physical examination, and blood tests, including a complete blood cell count and assessments of liver and renal function, until disease progression. Abdominal CT and measurements of tumor markers, such as carcinoembryonic antigen, carbohydrate antigen 199, carbohydrate antigen 125, carbohydrate antigen 153 and alpha fetoprotein, were done every 3 months.

A detailed description of the above data is shown in the CRF table. **Table 3** shows the test and data acquisition schedule for this experiment.

**Table 3:** The test and data acquisition schedule for this experiment.

Stage	Pre-operation	Intra-operation	Postoperation					Unplanned follow-up
Follow up period	14-1 days		0-12 month	13-24 month	25-36 month	37-48 month	49-60 month	
Baseline data collected	√	—	—	—	—	—	—	—
Inclusion and exclusion	√	—	—	—	—	—	—	—
Sign informed consent	√	—	—	—	—	—	—	—
Group determination	√	—	—	—	—	—	—	—
Fill in the basic information	√	—	—	—	—	—	—	—
Physical examination	√	—	√	√	√	√	√	—
Imaging examination	√	—	√	√	√	√	√	if necessary
Laboratory examination	√	—	√	√	√	√	√	—
Operation information	—	√	—	—	—	—	—	—
Postoperative pathology	—	√	—	—	—	—	—	if necessary
Safety observation	√	—	√	√	√	√	√	if necessary
Record adverse events	√	—	√	√	√	√	√	if necessary
Other works	√	√	√	√	√	√	√	if necessary

### Collection and Storage Management of Biochemical Specimens

For this study, blood samples, tissue samples of GC, and tissue samples of liver metastasis from the participants will be collected and subsequently tested by the laboratories and pathology departments. As previously reported (18), the collection and testing techniques for the above samples are quite mature. All blood samples will be destroyed after the test without preservation. GC specimens and liver metastatic tissue specimens will be sent to the pathological examination for timely preservation. They will be separated into cryopreservation tubes, and the separated tissues will be stored in liquid nitrogen tanks. They will be stored for five years and then destroyed six months after the end of the study.

### Evaluation of Technology Effectiveness

Contrast enhanced CT and tumor marker testing were performed in the first month after D2 gastrectomy and RFA treatment to evaluate the effectiveness of the technique and as a new baseline for future comparison. Every 2 to 4 months, additional CT examinations were performed to assess the progression of the disease. For patients who cannot be evaluated by enhanced CT, additional Hepatic contrast-enhanced ultrasonography, MRI or PET/CT can be used for further evaluation.

For the definition of effective rate of D2 gastrectomy, according to NCCN guidelines for diagnosis and treatment of gastric cancer, tumor clearance effectiveness is defined as that the tumor markers are not increased compared with the baseline data, and CT does not find the recurrence of new tumor or metastatic lymph nodes, or potential metastases in other parts and organs (19, 20).

For the definition of effective rate of RFA treatment, according to standardized terminology and reporting criteria for tumor ablation, technique effectiveness is defined as no evidence of residual tumor within 1 cm of the ablation defect; local tumor progression (LTP) is defined as any new peripheral or nodular enhancement within 1 cm or enlargement of the baseline ablation defect (21, 22).

### Sample Size Estimate and Statistical Analysis

The aim of REGATTA study (11) was to establish whether the addition of gastrectomy to standard chemotherapy improves survival among patients with advanced gastric cancer with a single non-curable factor. A single non-curable factor was defined as hepatic metastasis (H1; two to four lesions of maximum diameter ≤5 cm and minimum diameter ≥1 cm); peritoneal metastasis (P1) in the diaphragm or peritoneum caudal to the transverse colon without massive ascites or intestinal obstruction. However, our study was to compare the efficacy of D2 gastrectomy plus liver radiofrequency combined with chemotherapy versus chemotherapy alone in the treatment of advanced gastric cancer with unresectable synchronous liver metastases. Because there is a lack of international large-sample studies on the efficacy comparison of D2 gastrectomy plus liver radiofrequency plus postoperative chemotherapy versus chemotherapy alone, we can only estimate the sample size based on the correlation data according to the REGATTA study. The REGATTA study showed that adverse events occurred in 5 of 7 the patients with gastric cancer with liver metastases in chemotherapy group and 9 of 11 the patients with gastric cancer with liver metastases in chemotherapy plus gastrectomy group, which indicated that the incidence of adverse events in gastrectomy plus chemotherapy group was 60%, and that of chemotherapy group was 81.8%. Therefore, the incidence of adverse events in patients with gastric cancer with liver metastasis under the two treatment regimens in the REGATTA study was used to estimate the sample size.

To verify the comparison of the efficacy of D2 gastrectomy plus liver radiofrequency plus postoperative chemotherapy versus chemotherapy alone for patients with GCLM, we designed a superiority study with a superiority margin of 5% (α= 0.05, β= 0.20, 80% power). With a standard error of 0.05 and a confidence interval of 80%, a sample size of 178 is necessary. To minimize sampling error and account for the rate of loss to follow-up for various reasons, we determined the sample size to be 200 participants. Standard descriptive statistics will be used to analyze qualitative and quantitative variables such as relative and absolute frequencies, frequency tables, means, medians, standard deviations, ranges, and quartiles. A 95% confidence level will be considered appropriate for analysis. Descriptive statistics will also be used to describe the most relevant clinical parameter measurements. Analysis of categorical variables will be performed by two-sample t tests or Fisher’s exact test. If there is no significant difference in the response rate to D2 gastrectomy plus liver radiofrequency plus postoperative chemotherapy versus chemotherapy alone, then the result will be negative, that is, the difference is not related to whether to apply the treatment strategy of D2 gastrectomy plus liver radiofrequency plus postoperative chemotherapy for patients with GCLM; otherwise, there is a relationship.

### Study Endpoints

In this study, the primary endpoint will be the OS. The secondary endpoints PFS and total complication rate. The total complications will include postoperative and chemotherapy complications. Postoperative complications were defined as events occurring within 30 days after the procedure, the severity of which was assessed by the ClavienDindo classification system (23, 24). Postoperative complications will include surgical site infection (SSI refers to the infection that occurs in the incision, deep organ or cavity during the perioperative period. It is mainly divided into superficial wound tissue infection, deep wound tissue infection and organ/cavity infection); anastomotic leaks and duodenal blow-out (identified clinically or radiographically); Chylous fistula (defined as milky white liquid in peritoneal drainage fluid after the start of enteral nutrition); respiratory complications (defined as clinical manifestation of pneumonia or bronchopneumonia confirmed by computed tomographic scan); and other complications (such as: delayedgastricemptying, intestinal obstruction, anastomotic and/or abdominal bleeding, abdominal abscess, pancreatic fistula, pancreatitis and so on.). Postoperative mortality was defined as all-cause death occurring within 30 days after the procedure. Chemotherapy complications refer to the side effects of chemotherapy, such as ototoxicity and nephrotoxicity. For those patients delayed postoperative systemic radiotherapy and/or chemotherapy after 4 weeks of surgery due to surgery and its complications, their clinical data were recorded in detail and analyzed by subgroup stratification.

Adverse events are adverse medical events that occur after surgical and medical treatment in patients in clinical trials. Adverse events, whether treatment-related or not, will be considered from the date participants sign informed consent until 5 years after the end of treatment in this study. The nature and severity of adverse events will be assessed in accordance with “expert consensus on diagnostic criteria for postoperative complications of gastrointestinal cancer in China”. The investigator will determine if the adverse event is clinically significant, and if so, it will be identified as an adverse event. The investigators will assess possible relationships among the adverse events, the investigational drugs and the surgery to assess adverse events and their causal relationship to treatment. Adverse reactions include those results recorded as positive, correlated, and possibly correlated. The investigators will keep a full record of serious adverse events from the start of the surgery to the end of the study. All serious adverse events will be reported regardless of whether they are related to the use of drugs or surgery in the study. It is the responsibility of the investigators to notify independent ethics committees or governmental regulatory authorities related to adverse events.

### Follow-up

When patients are discharged from the hospital, the first follow-up is to be performed on the 14th day after surgery. The patients will be given physical examination, imaging examination and laboratory examination to determine the postoperative complications of the patients during the follow-up period. The next chemotherapy plan will be formulated for the patients according to the above results. Physical examination and laboratory examination will be performed at least monthly on all enrolled patients. Imaging examinations will be performed every three months. The patients will be followed up by researchers every month to every 3 months. In addition to the above, the complications of chemotherapy will be examined. Recurrence, metastasis, death, and dates of patients will be recorded. The contents of each follow-up will be collated and combined. The follow-up contents will be recorded in **Table 3**.

### Patient Protection/Written Informed Consent Forms

Both parties will ensure the protection of the patient’s personal records. Except for documents where it is required by law, patient names will not be included in any form in tabular reports, publications, or any type of research-related document. Informed consent will be formulated in strict accordance with Chinese laws and regulations. Written informed consent, including all changes made throughout the study, must be preapproved by the IRB/ICB before inclusion in the study. Medical staff will obtain a signature with written informed consent from each patient (if the patient is unable to make their own decision for various reasons, the immediate family will decide on their behalf) prior to any specific activities related to the study. Researchers will submit and keep original copies of all written informed consent forms signed by patients and provide additional copies to patients or their immediate family members for their records.

### Monitoring of the Study

Before the start of the study, the personnel of the project unit will visit all of the research centers and discuss with the researcher (and/or other research-related personnel) the responsibility of the researcher for the research program and the responsibility of the project undertaking unit or representative.

During the study period, the project undertaker or the supervisor representing the project undertaker will regularly contact the research center for a number of reasons, including the following: providing information and technical support; establishing randomized grouping as required; confirming that the investigator complies with the study plan, that the data on the CRFs are accurately recorded, and the dosage of drugs being used is checked; and carrying out original data analysis (e.g., the data on CRFs are related to the records of patients in the hospital, and the research will compare these with other records). This requires direct access to the original records of each patient (e.g., clinical charts).

The interim analysis will be performed when half of the planned sample size is enrolled. The prespecified stopping criteria in the study protocol are as follows: if D2 gastrectomy plus liver radiofrequency combined with chemotherapy is superior to chemotherapy alone, and the analysis is statistically significant, study termination will be considered, and subsequent patients will receive D2 gastrectomy plus liver radiofrequency combined with chemotherapy. However, if the efficacy of D2 gastrectomy plus liver radiofrequency combined with chemotherapy is not superior to chemotherapy alone, and the analysis results are statistically significant, the study will be considered as invalid and the subsequent enrolled patients will receive chemotherapy alone. The Data and Safety Monitoring Committee of the China Clinical Trial Registry independently reviewed the interim analysis protocol and may decide to terminate the study early with the approval of the ethics committee of each center.

Representatives authorized by project undertakers, regulatory departments, and independent ethics committees may visit the center for inspections, including verifying the original data. The purpose of the inspections of the site and personnel is to systematically and independently examine all research-related behaviors and documents, to determine that these behaviors have been managed and that the data have been analyzed, recorded, and accurately reported in accordance with the research program, GCP, ICH guidelines and other regulatory requirements.

### Patient and Public Involvement

Patients and the public will not participate in the design, implementation, dissemination, or reporting of our studies.

### Ethical Approval and Consent to Participate

This trial is a prospective multicenter randomized controlled study designed to explore the best strategy for the treatment of gastric cancer with unresectable synchronous liver metastasis. This study will strictly abide by all legal requirements, regulations and general principles formulated by international agencies concerning ethical conduct in human biomedical research and by the Declaration of Helsinki and the International Ethical Guidelines for Biomedical Research Involving Human Beings. This study protocol was approved by the Chinese Registered Clinical Trial Ethics Committee (Hong Kong Center, China Clinical Trial Registry, Kowloon Pond Baptist University Road, Hong Kong Special Administrative Region, China, Approval No. ChiECRCT20200331, Resolution 15 November 2020). The ERC is obliged to evaluate the progress of the study periodically. As soon as any adverse event (AES) occurs, the relevant information will be reported to the IRB.

## Discussion

At present, chemotherapy, as a treatment strategy for patients with GCLM, has been recommended as the standard treatment for such patients by Japanese, American and international guidelines (4, 5). With the continuous improvement of chemotherapy schemes, the prognosis of patients with GCLM has been improved to a certain extent (7, 25). A phase 3, randomized controlled trial (11), conducted in 2016, showed that compared to chemotherapy alone, chemotherapy after gastrectomy did not show any survival benefit. However, Fujitani et al. performed D1 lymphadenectomy in gastrectomy for primary gastric cancers and did not remove metastatic lesions in livers in their study. Therefore, whether more thorough D2 lymphadenectomy in gastrectomy for primary gastric cancers plus radiofrequencies for liver metastases plus postoperative chemotherapy can improve the prognosis of patients with GCLM deserves further high-level evidence-based medical evidence.

However, there remains a lack of multicenter randomized controlled trials on D2 gastrectomy plus liver radiofrequency plus postoperative chemotherapy versus chemotherapy alone. The aim of the study was to establish a multicenter randomized controlled trial to evaluate the efficacy of D2 gastrectomy plus liver radiofrequency plus postoperative chemotherapy versus chemotherapy alone. OS and PFS will be taken as the main indicators to study a comparison of the efficacy of two treatment strategies for GCLM in this study. This study will be the first prospective multicenter randomized controlled study comparing the efficacy of D2 gastrectomy plus liver radiofrequency plus postoperative chemotherapy versus chemotherapy alone in China, which may change the selection of standard treatment strategies for GCLM worldwide.

If the results of this study meet our expectations, it will be a great encouragement for health care providers to improve the prognosis of patients with GCLM. We hope to collect lessons and suggestions from this study and integrate them into clinical practice to improve clinical treatments, to further improve the quality of life, and to change the standard treatment strategy for these patients.

## Data Availability Statement

The original contributions presented in the study are included in the article/supplementary material. Further inquiries can be directed to the corresponding authors.

## Ethics Statement

The studies involving human participants were reviewed and approved by Chinese Clinical Trial Registry. The patients/participants provided their written informed consent to participate in this study.

## Author Contributions

Concept Proposal: XL. Survey and Data Summary: WW. Data Collection, analysis and statistics: WW, RG, PY, DD, and ZM. Specific scheme implementation GJ, XL, PY, DD, ZM, XY, RG, and WW. Research Regulatory: GJ and XL. Writing – Draft: WW. Writing-Proofreading and Editing: XL and WW. All authors have approved the final version of this manuscript.

## Funding

This work is supported by grants from the National Natural Science Foundation of China (Key Program 82100680) by PFY and GJ. This work is also supported by grants from the Shaanxi Innovation Team(2021-TD-43) by XL and GJ.

## Conflict of Interest

The authors declare that the research was conducted in the absence of any commercial or financial relationships that could be construed as a potential conflict of interest.

## Publisher’s Note

All claims expressed in this article are solely those of the authors and do not necessarily represent those of their affiliated organizations, or those of the publisher, the editors and the reviewers. Any product that may be evaluated in this article, or claim that may be made by its manufacturer, is not guaranteed or endorsed by the publisher.

## Abbreviations

GC, gastric cancer; GCLM, advanced gastric cancer with unresectable synchronous liver metastasis; RCT, randomized controlled study; WHO, World Health Organization; CRF, Case Report Form; EMR, endoscopic mucosal resection; PS, performance status; RFA, radiofrequency ablation; PFS, progression-free survival; OS, overall survival.
